# A Rare Case of Multifocal Prostatic Blue Nevus

**DOI:** 10.1155/2018/7820717

**Published:** 2018-02-21

**Authors:** Elias J. Farran, Preston S. Kerr, Christopher D. Kosarek, Joseph Sonstein, Eduardo J. Eyzaguirre

**Affiliations:** ^1^Division of Urology, Department of Surgery, University of Texas Medical Branch, 301 University Blvd., Galveston, TX, USA; ^2^Department of Pathology, University of Texas Medical Branch, 301 University Blvd., Galveston, TX, USA

## Abstract

Prostatic blue nevus is a rare benign pathologic diagnosis most commonly diagnosed incidentally on many different types of prostate specimens. Blue nevus is the deposition of stromal melanin characterized by spindle cells within the fibromuscular stroma which stains positive for melanin-specific stains Fontana-Masson and S100 and stains negative for CD68, HMB45, and iron stains. We report the case of a multifocal and bilateral blue nevus in a 52-year-old Hispanic male who presented with an elevated prostate-specific antigen of 4.3 and mild obstructive lower urinary tract symptoms, found by transrectal ultrasound-guided prostate needle biopsy. The biopsy also revealed benign prostatic tissue with postatrophic hyperplasia and chronic inflammation. This is the 35th reported case of prostatic blue nevus and the third to show multifocal blue nevus.

## 1. Introduction

Melanocytic lesions are an often-overlooked pathologic process that occurs in the prostate. Of these extremely rare lesions, the most commonly found is the prostatic blue nevus, also known as pigmented melanocytosis or prostatic pigmentary nevohyperplasia [1]. Blue nevus is the deposition of stromal melanin characterized by spindle cells within the fibromuscular stroma which stains positive for melanin-specific stains Fontana-Masson and S100, while it stains negative for CD68 proteins, HMB45, and iron stains [1]. Blue nevus is asymptomatic and benign and has been incidentally diagnosed following prostatectomy (11 cases), transurethral resection of the prostate (TURP, 6 cases), autopsy (5 cases), and transrectal ultrasound-guided prostate needle biopsy (TRUS PNBx, 2 cases) (Table 1).

## 2. Case Presentation

**Figure 1:**
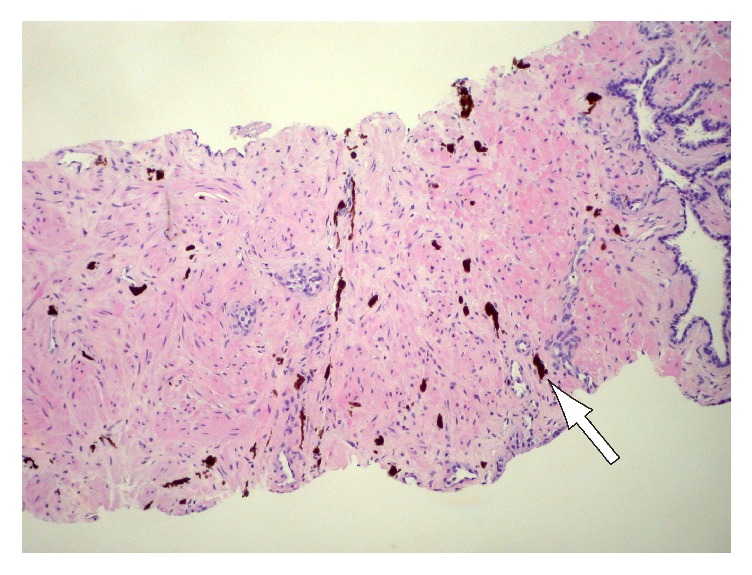
Low-power view of blue nevus in the prostate as a cluster of pigmented spindle to round cells in the stroma (hematoxylin-eosin, original magnification: ×10).

**Figure 2:**
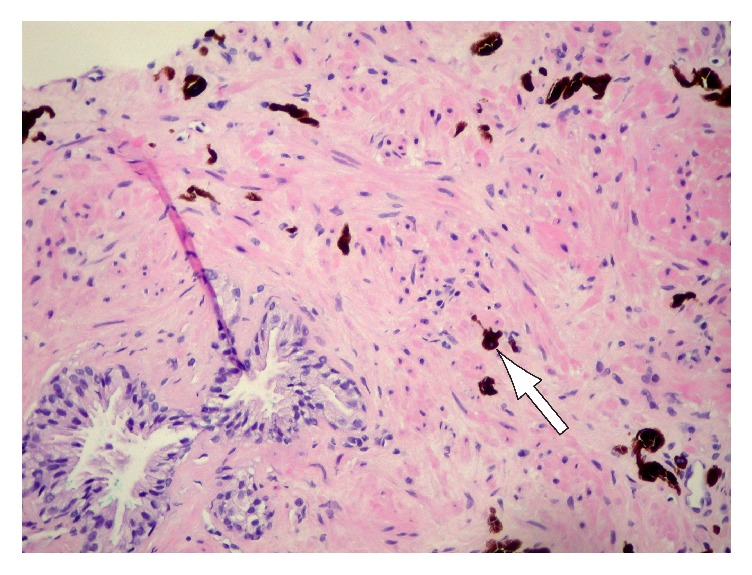
Individual heavily pigmented spindle cells distributed in between prostatic stroma and glands (hematoxylin-eosin, original magnification: ×20).

A 52-year-old healthy Hispanic male presented to an outpatient urology clinic with an elevated prostate-specific antigen (PSA) of 4.1 along with mild obstructive lower urinary tract symptoms. There was no family history of prostate cancer. The physical examination including the digital rectal examination was unremarkable. The patient was seen again 3 months later with a PSA of 4.3 and after discussion with the patient he elected to undergo a 12-core TRUS PNBx. The following month when the biopsy was performed, the PSA had slightly decreased to 3.4. The prostate was visualized in the sagittal and transverse planes via ultrasound probe and was unremarkable. Volume was measured to be 33 cm^3^ (PSA density of 0.10 ng/mL/g). Final pathology demonstrated blue nevus in one out of six cores on the right and two out of six cores on the left. On microscopic analysis with hematoxylin-eosin stain, individual heavily pigmented spindle cells distributed in between prostatic stroma and glands were noted (Figures 1 and 2). The remaining specimen consisted of benign prostatic tissue with postatrophic hyperplasia and chronic inflammation. The patient's voiding symptoms improved with terazosin and no further workup was undertaken. The patient is now being followed up for routine prostate cancer surveillance as per the American Urological Association (AUA) guidelines [26].

## 3. Discussion

Blue nevus is a rare lesion of dermal melanocytes most commonly found in the skin, but it has been reported in the oral mucosa, sclera, cervix, vagina, and prostate [27]. The appearance of this lesion in nonintegumentary tissues is not fully understood; the prevailing hypothesis is that melanoblasts originate in the neural crest and migrate with the mesoderm into connective tissue, where they remain latent until maturing into melanocytes [28]. Proliferation induced by inflammation or other insults of these latent melanoblasts can explain acquired cases of blue nevi [29]. An alternative hypothesis proposes development from the neoplastic growth of Schwann cells of dermal nerves which became melanogenetic as they proliferated [30].

Blue nevus grossly appears as multiple brown to black streaks or nodules that range in size from 0.1 cm to 2.0 cm [1]. Microscopically, prostatic blue nevus consists of stromal cells that contain finely granular brown or black pigment, which may also be seen in the extracellular matrix [11]. The cells can extensively infiltrate the surrounding fibromuscular stroma individually or as irregularly clustered collections [4]. The pigment-laden cells are usually spindle in shape with bipolar, elongated dendritic cytoplasmic processes but can also be round, ovoid, or polygonal (Figures 1 and 2). The nuclei have been described as centrally located and often obscured by the abundant melanin present in the cytoplasm [2]. It is also important to recognize the benign nature of these lesions and not confuse them with more aggressive melanocytic lesions of the prostate such as malignant melanoma. Hypercellularity, diffuse atypia, increased mitotic activity, and positive immunostaining for HMB45 should help in differentiating malignant melanoma from blue nevus.

## 4. Conclusion

Review of the literature indicates that blue nevus typically presents as a single focus and is characteristically diagnosed on TURP and prostatectomy specimens. Although no risk factors for blue nevus have been identified, our discovery of just the second case in a Hispanic male may suggest variability in risk among different races/ethnicities [23]. Of the other 34 reported cases of blue nevus, only two have shown multifocal blue nevus [24, 25]. Diagnosis is most often made on prostatectomy or TURP specimens; however, there have been two reported cases documenting diagnosis by TRUS PNBx, making this the third reported case [18, 19]. As in all other cases, blue nevus presented in an asymptomatic fashion. The importance of this case lies in the rarity of such a diagnosis as it is highly likely that both urologist and pathologist alike have not come across such a diagnosis. The recognition of the benign nature of blue nevus and multifocal blue nevus need to be emphasized as further workup and surveillance outside of routine prostate cancer screening carries no benefit. As always, all routine prostate cancer screening should follow the shared decision-making mantra endorsed by the AUA [26].

## Figures and Tables

**Table 1:** Literature review of blue nevus cases.

Source, year	Procedure	Age (years)	Race	Extent
Nigogosyan et al. [2], 1963	Autopsy	50	NA	Focal

Guillan and Zelman [3], 1970	Autopsy	NA	NA	Focal

Jao et al. [4], 1971	Prostatectomy	76	W	Focal

Gardner and Spitz [5], 1971	Autopsy	20	AA	Focal

Block et al. [6], 1972	Prostatectomy	66	W	Focal

Langley and Weitzner [7], 1974	NA	NA	W	Focal

Tannenbaum [8], 1974	NA	NA	NA	Focal

Rios and Wright [9], 1976	Autopsy	67	AA	Focal

Kovi et al. [10], 1977	TURP	65	AA	Focal

Ro et al. [11], 1988	TURP	68	AA	Focal
	TURP	76	W	Focal

Botticelli et al. [12], 1989	Prostatectomy	69	W	Focal
	Prostatectomy	70	W	Focal
	Prostatectomy	66	NA	Focal

Lew et al. [13], 1991	Prostatectomy	80	AA	Focal

Martinez Martinez et al. [14], 2017	Prostatectomy	81	NA	Focal
	Prostatectomy	69	NA	Focal

Vesga et al. [15], 1995	NA	NA	NA	Focal

Redondo Martínez et al. [16], 1998	TURP	58	NA	Focal

Cuervo Pinna et al. [17], 2001	Prostatectomy	71	NA	Focal

Di Nuovo et al. [18], 2002	Needle Biopsy	66	NA	Focal

Humphrey [19], 2003	Needle Biopsy	70	NA	Focal

Anderco et al. [20], 2010	TURP	69	NA	Focal

Kudva and Hegde [21], 2010	TURP	53	NA	Focal

Raspollini et al. [22], 2011	Prostatectomy	64	NA	Focal

Montalvo and Redrobán [23], 2013	Prostatectomy	63	H	Focal

Ponte et al. [24], 2014	Prostatectomy	69	W	Multifocal

Ponte et al. [25], 2016	Prostatectomy	74	NA	Multifocal

Present report	Needle biopsy	52	H	Multifocal

AA, African American; H, Hispanic; W, non-Hispanic White, TURP, transurethral resection of the prostate.

## References

[1] Dailey V. L., Hameed O. (2011). Blue nevus of the prostate. *Archives of Pathology & Laboratory Medicine*.

[2] Nigogosyan G., De La Pava S., Pickren J. W., Woodruff M. W. (1963). Blue nevus of the prostate gland. *Cancer*.

[3] Guillan R. A., Zelman S. (1970). The Incidence and Probable Origin of Melanin in the Prostate. *The Journal of Urology*.

[4] Jao W., Fretzin D. F., Christ M. L., Prinz L. M. (1971). Blue nevus of the prostate gland. *Archives of Pathology*.

[5] Gardner W. A., Spitz W. U. (1971). Melanosis of the Prostate Gland. *American Journal of Clinical Pathology*.

[6] Block N. L., Weber D., Schinella R. (1972). Blue Nevi and Other Melanotic Lesions of the Prostate: Report of 3 Cases and Review of the Literature. *The Journal of Urology*.

[7] Langley J. W., Weitzner S. (1974). Blue Nevus and Melanosis of Prostate. *The Journal of Urology*.

[8] Tannenbaum M. (1974). Differential diagnosis in uropathology III. Melanotic lesions of prostate: Blue nevus and prostatic epithelial melanosis. *Urology*.

[9] Rios C. N., Wright J. R. (1976). Melanosis of the Prostate Gland: Report of a Case with Neoplastic Epithelium Involvement. *The Journal of Urology*.

[10] Kovi J., Jackson A. G., Jackson M. A. (1977). Blue nevus of the prostate: ultrastructural study. *Urology*.

[11] Ro J. Y., Grignon D. J., Ayala A. G., Hogan S. F., Tetu B., Ordonez N. G. (1988). Blue nevus and melanosis of the prostate. Electron-microscopic and immunohistochemical studies. *American Journal of Clinical Pathology*.

[12] Botticelli A. R., Di Gregorio C., Losi L., Fano R. A., Manenti A. (1989). Melanosis (pigmented melanocytosis) of the prostate gland. *European Urology*.

[13] Lew S., Richter S., Jelin N., Siegal A. (1991). A blue naevus of the prostate: a light microscopic study including an investigation of S‐100 protein positive cells in the normal and in the diseased gland. *Histopathology*.

[14] Martinez Martinez C., Garcia Gonzalez R., Castañeda Casanova A. (2017). Blue Nevus of the Prostate: Report of Two New Cases with Immunohistochemical and Electron-Microscopic Studies. *European Urology*.

[15] Vesga Molina F., Acha Pérez M., Llarena Ibarguren R., Pertusa Peña C. (1995). Intraprostatic blue nevus. *Archivos Españoles de Urología*.

[16] Redondo Martínez E., Rey López A., Díaz Cascajo C. (1998). Blue nevus of the prostate. Differential diagnosis of prostatic pigmented lesions. *Archivos Españoles de Urología*.

[17] Cuervo Pinna C., Godoy Rubio E., Parra Escobar J. L., Sánchez Blasco E., Valverde Valverde J., Moreno Casado J. (2001). Prostatic blue nevus. Terminology standardization of prostatic pigmented lesions. *Actas Urológicas Españolas*.

[18] Di Nuovo F., Sironi M. G., Spinelli M. (2002). True prostatic blue nevus associated with melanosis: case report, histogenesis and review of the literature. *Advances in Clinical Pathology*.

[19] Humphrey P. A. (2003). *Prostate Pathology*.

[20] Anderco D., Lazăr E., Taban S., Miclea F., Dema A. (2010). Prostatic blue nevus. *Romanian Journal of Morphology and Embryology*.

[21] Kudva R., Hegde P. (2010). Blue nevus of the prostate. *Indian Journal of Urology*.

[22] Raspollini M. R., Masieri L., Tosi N., Santucci M. (2011). Blue nevus of the prostate: incidental finding in radical prostatectomy specimen with a pre-operative echographic image of peripheral hypoechogenic nodule. *Archivio Italiano di Urologia e Andrologia*.

[23] Montalvo N., Redrobán L. (2013). Unusual histopathological diagnosis of prostatic blue nevus: a case report. *Journal of Medical Case Reports*.

[24] Ponte R., Ravetti J. L., Pacella M., Toncini C. (2014). Multifocal blue nevus of the prostate: a case report. *Analytical and Quantitative Cytology and Histology*.

[25] Ponte R., Ravetti J. L., Calamaro P., Pacella M., Toncini C. (2016). Another case of multifocal blue nevus of the prostate. *Analytical and Quantitative Cytology and Histology*.

[26] Carter H. B. (2013). American urological association (AUA) guideline on prostate cancer detection: Process and rationale. *BJU International*.

[27] Craddock K. J., Bandarchi B., Khalifa M. A. (2007). Blue nevi of the Müllerian tract: case series and review of the literature. *Journal of Lower Genital Tract Disease*.

[28] Ahmad M., Reams W. M. (1978). The development of melanoblasts from leg bud mesenchyme grown in the celom of chick embryos. *Annals of Anatomy*.

[29] Hori Y., Kawashima M., Oohara K., Kukita A. (1984). Acquired, bilateral nevus of Ota-like macules. *Journal of the American Academy of Dermatology*.

[30] Nakai T., Rappaport H. (1963). A study of the histogenesis of experimental melanotic tumors resembling cellular blue nevi: The evidence in support of their neurogenic origin. *The American Journal of Pathology*.

